# The Pakistan National Emergency Department Surveillance Study (Pak-NEDS): Introducing a pilot surveillance

**DOI:** 10.1186/1471-227X-15-S2-S1

**Published:** 2015-12-11

**Authors:** Mohammed Umer Mir, Abdulgafoor M Bachani, Haseeb Khawaja, Shiraz Qayoom Afridi, Sabir Ali, Muhammad Mujeeb Khan, Seemin Jamali, Fareed Ahmed Sumalani, Adnan A Hyder, Junaid A Razzak

**Affiliations:** 1 Department of Emergency Medicine, Aga Khan University, Stadium Road, Karachi, Pakistan; 2 Johns Hopkins International Injury Research Unit, Health Systems Program, Department of International Health, Johns Hopkins Bloomberg School of Public Health, Baltimore, Maryland, USA; 3 Shifa International Hospital, Islamabad, Pakistan; 4 Lady Reading Hospital, Peshawar, Pakistan; 5 Mayo Hospital, Lahore, Pakistan; 6 Benazir Bhutto Hospital, Rawalpindi, Pakistan; 7 Jinnah Postgraduate Medical Centre, Karachi, Pakistan; 8 Civil Hospital, Quetta, Pakistan; 9 Department of Emergency Medicine, Johns Hopkins School of Medicine, Baltimore, Maryland, USA; 10 The author was affiliated with the Department of Emergency Medicine, Aga Khan University, Karachi, Pakistan at the time when study was conducted

**Keywords:** Emergency department, surveillance, mulit-center, methodology, Pakistan

## Abstract

**Background:**

Evidence-based decision making is essential for appropriate prioritization and service provision by healthcare systems. Despite higher demands, data needs for this practice are not met in many cases in low- and middle-income countries because of underdeveloped sources, among other reasons. Emergency departments (EDs) provide an important channel for such information because of their strategic position within healthcare systems. This paper describes the design and pilot test of a national ED based surveillance system suitable for the Pakistani context.

**Methods:**

The Pakistan National Emergency Department Surveillance Study (Pak-NEDS) was pilot tested in the emergency departments of seven major tertiary healthcare centres across the country. The Aga Khan University, Karachi, served as the coordinating centre. Key stakeholders and experts from all study institutes were involved in outlining data needs, development of the study questionnaire, and identification of appropriate surveillance mechanisms such as methods for data collection, monitoring, and quality assurance procedures. The surveillance system was operational between November 2010 and March 2011. Active surveillance was done 24 hours a day by data collectors hired and trained specifically for the study. All patients presenting to the study EDs were eligible participants. Over 270,000 cases were registered in the surveillance system over a period of four months. Coverage levels in the final month ranged from 91-100% and were highest in centres with the least volume of patients. Overall the coverage for the four months was 79% and crude operational costs were less than $0.20 per patient.

**Conclusions:**

Pak-NEDS is the first multi-centre ED based surveillance system successfully piloted in a sample of major EDs having some of the highest patient volumes in Pakistan. Despite the challenges identified, our pilot shows that the system is flexible and scalable, and could potentially be adapted for many other low- and middle-income settings.

## Background

Although the use of data for prioritization of healthcare programs and services is often touted as the cornerstone of sound decision-making in public health, in many low- and middle-income countries (LMICs), this is simply not the case[1-3]. This is not entirely because policymakers, health officers, or program planners are averse to making evidence based decisions, but partly because the necessary information is not available. This has, however, been changing in recent years, and there is more of a demand for such data globally.

Emergency departments (EDs) are located at the boundary between hospitals and the communities that they serve [4]. They cater to a wide spectrum of health issues, and in many cases, maximize access to specific healthcare services for all sections of the community [5]. This strategic positioning of the EDs in the healthcare system can allow them to play key roles in areas such as monitoring health care access of communities, surveillance of communicable and non-communicable diseases (including injuries), providing preventive services to the community (in addition to acute care e.g. smoking cessation counseling for smokers presenting with coronary heart disease), and in evidence based policy development [4,6,7]. Surveillance information from these departments can be used to assess the patterns of disease and acute health needs. Such information can be used: to better respond to rapidly developing public health emergencies; to better understand the linkages between environmental events and ED visits; to understand the prevalence of injuries requiring medical attention in the community; and for quality management of services within healthcare facilities [8]. Several high-income countries (HICs) have taken advantage of the unique position of EDs and established surveillance systems for different needs, such as the National Electronic Injury Surveillance System (NEISS) in the United States (US) which was successfully used to identify an injury outbreak (due to all-terrain vehicles - ATVs) which led to a targeted national intervention [9,10]. Other examples of successful ED-based public health surveillance systems are the Weapons Reporting Injury Surveillance System (WRISS) in Massachusetts, and the EMERGEncy Net ID [11,12]. Similar efforts have been underway recently in the developing world such as the Road Crash Victim Information System (RCVIS) in Cambodia that attempts to collate data on road traffic injuries from different sources [13], and the Road Traffic Injury Surveillance System in Karachi, Pakistan [14].

In LMICs, however, the potential of EDs as a source of vital information to guide and improve the provision of healthcare services, and public health in general, has not been fully harnessed. Studies from Pakistan have shown poor pre-hospital and facility-based care systems [15], which have largely been attributed to a lack of local evidence to inform these systems [16]. Even basic information on types of patients seen or discharged from EDs at the national level is not usually available in Pakistan and many other LMICs. A national ED-based surveillance system can play a pivotal role in providing information for local hospital decision-makers as well as for health planners at the regional and national level. Evidence based decisions about resource allocation, including material, human, and financial resources can then be made.

In this paper, we describe the development and pilot test of the Pakistan National Emergency Department Surveillance System (Pak-NEDS) in seven major tertiary healthcare centers across Pakistan including the development of data collection tools, methods, and processes.

## Conceptual framework

**Figure 1:**
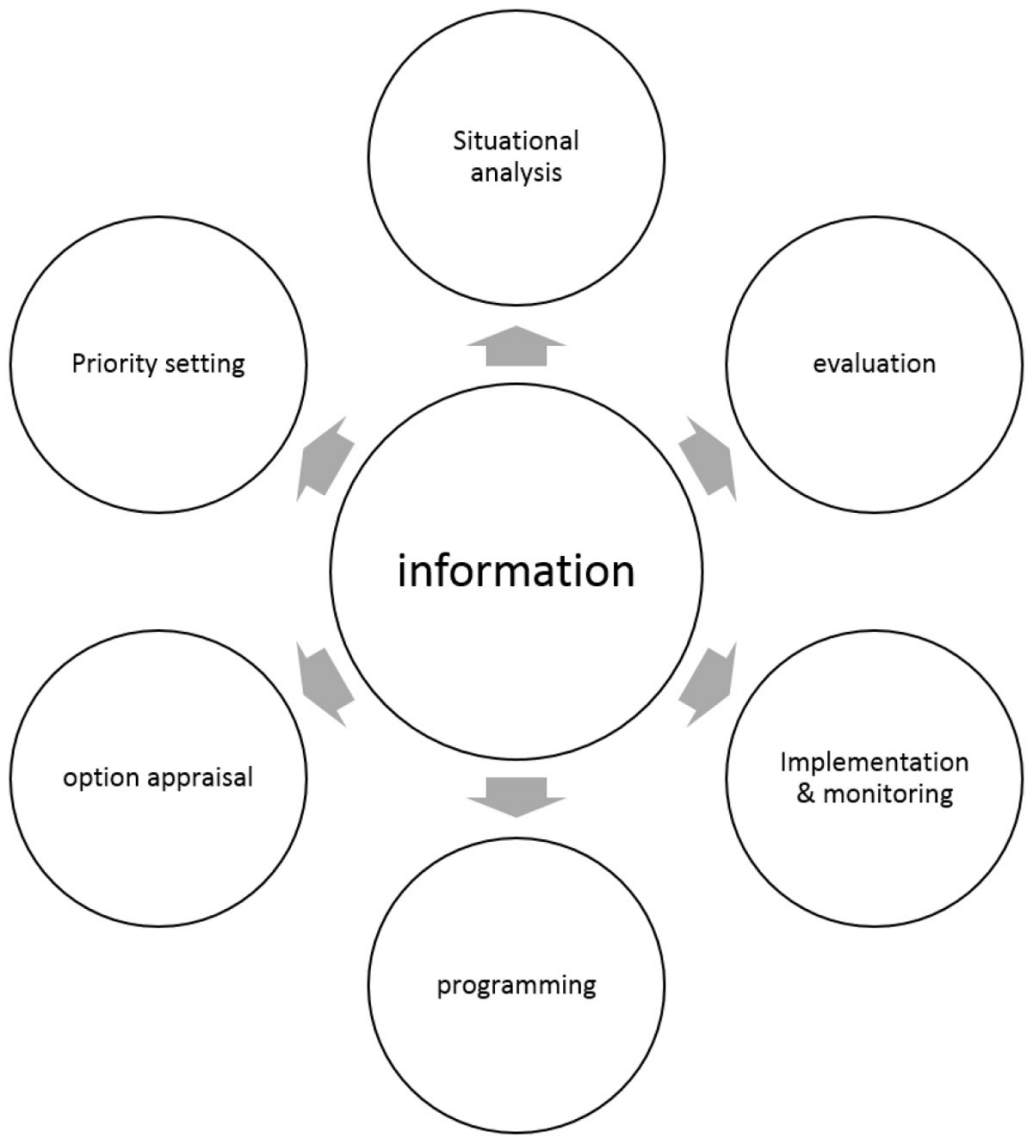
**Information use in health management **[19].

Information gained from health information systems supports evidence based decision-making at multiple levels (Figure 1) and is considered a significant tool for effective health management [17,18]. It is used by managers for situation analysis, priority setting, and evaluations of implemented interventions or programs. Physicians and other health care providers can utilize this information for patient management. Policy makers work with the evidence to inform health policies affecting the overall health system.

**Figure 2:**
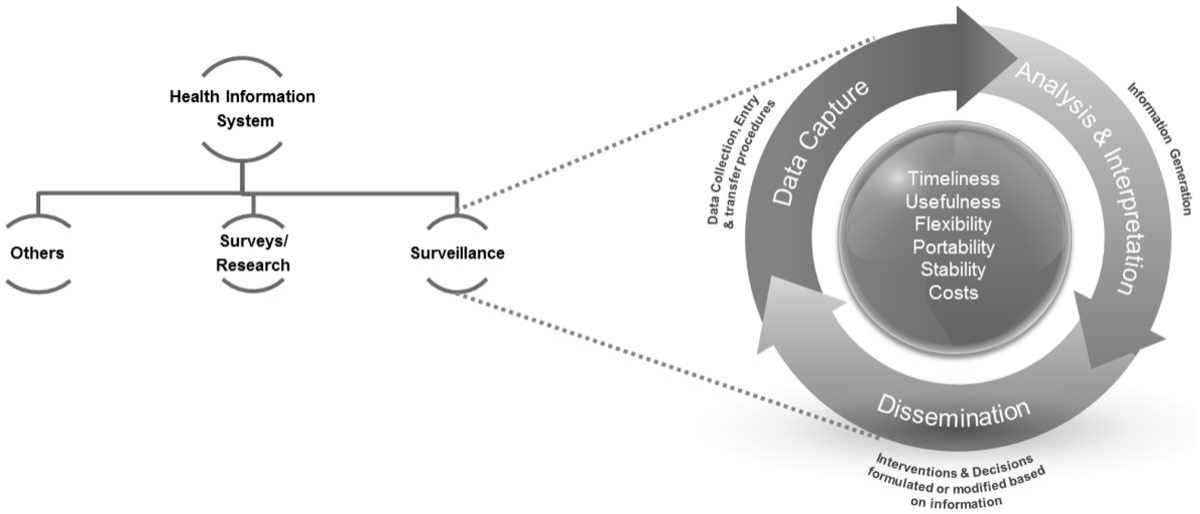
**Health information system & Surveillance**.

Health information systems can be considered to have subunits or 'subsystems' gathering information from different sources including the health sector, surveys and research, and other sectors such as census bureaus or departments of transport and safety, department of labor, etc. [19]. An important component of the health sector information subsystem is surveillance systems, monitoring disease or patient patterns and operating at the institutional level, e.g. in hospitals or in communities. The more obvious components of such systems include data collection, transmission, processing, and analysis (Figure 2).

Timely collection and processing, and analysis of valid information are important elements of any surveillance system. Other qualities are also essential if these systems are contextualized within the larger health information system and the health system itself, as proposed by the Centers for Disease Control and Prevention (CDC) [20]. *Usefulness *is maximized by considering the different consumers and the utility of the information generated by the surveillance system. The surveillance system not only needs to generate information satisfying local data needs, but also needs to complement the overall health information system for it to be an effective tool for use in health systems planning and policymaking. The system needs to be *flexible *so that it may adjust for changing needs over time. It should be *acceptable *to all stakeholders including the consumers of the information, in order to ensure validity of the information produced from the system and for maximal uptake by the intended users. The system needs to be *portable *for it to be replicated in other settings, which is generally accomplished with simpler systems. Portability contributes to standardization and coalescing capacity for surveillance systems in different locations to form regional or national databases. Other considerations include system *stability *and *costs*. Stability can be gauged by the uninterrupted operation of such systems over time under varying conditions, while costs can be analyzed using economic evaluations such as a cost-benefit analysis. All of these factors are vital for the long-term sustainability of the system [19,20]. Pak-NEDS was developed in consideration of these principles and how they could be maximally adhered to in the Pakistani context.

## Methods

### Setting

**Figure 3:**
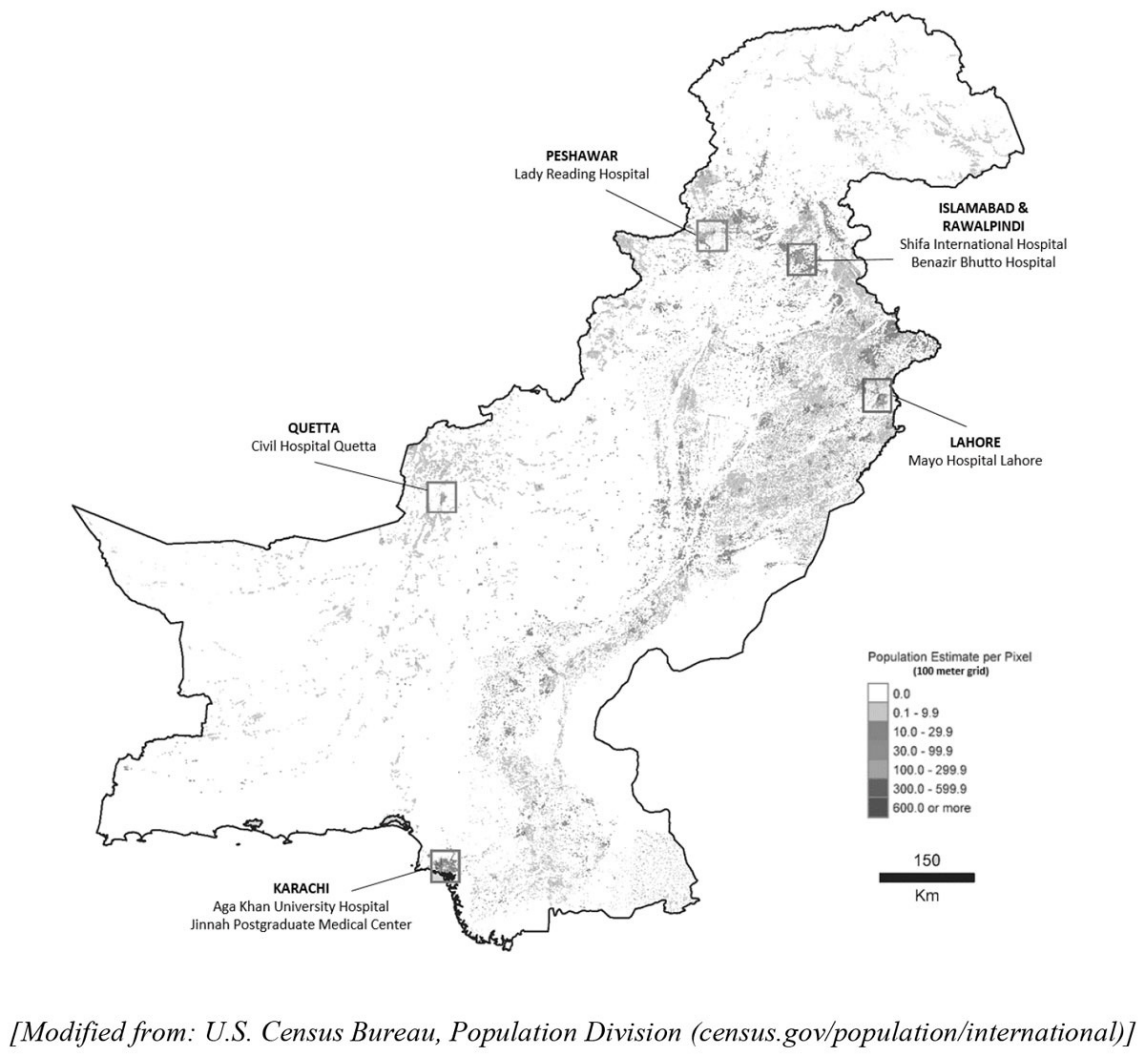
**Map of Pakistan**.

Seven major tertiary care centers from across the country were selected for the study (Table 1). The Jinnah Postgraduate Medical Center (JPMC) (Karachi), Civil Hospital (Quetta) (CHQ), Mayo Hospital (Lahore) (MHL), and Lady Reading Hospital (LRH) (Peshawar), are all public institutions located in the four provincial capitals (Figure 3). Other institutes were Shifa International Hospital (SIH), a private hospital in the national capital of Islamabad, and the Benazir Bhutto Hospital (RGH) in Rawalpindi. The coordinating center for the surveillance system was the Aga Khan University (AKU, a private tertiary care institute in Karachi, Pakistan). Inclusion of these hospitals ensured representation from all four socio-culturally diverse provinces of the country, and from both the public and private healthcare sectors. The populations served by these EDs include the cities and surrounding smaller towns and rural centers.

**Table 1:** Study centers selected for pilot testing of Pakistan National Emergency Department Surveillance (Pak-NEDS)

Institution	Benazir Bhutto Hospital (BBH)	Lady Reading Hospital (LRH)	Jinnah Post-graduate Medical Center (JPMC)	Mayo Hospital Lahore (MHL)	Civil Hospital Quetta (CHQ)	Shifa International Hospital (SIH)	Aga Khan University Hospital (AKU)
**Location**	Rawalpindi	Peshawar	Karachi	Lahore	Quetta	Islamabad	Karachi
**Type**	Tertiary Care - Public	Tertiary Care - Public	Tertiary Care - Public	Tertiary Care - Public	Tertiary Care - Public	Tertiary Care - Private	Tertiary Care - Private
**Coverage (%)**							
**- Final Month**	91	92	97	97	98	100	100
**- Overall**	76	79	95	89	97	90	91
**Cases (N)**	63,249	59,914	52,550	46,755	35,325	4,589	12,054

**Figure 4:**
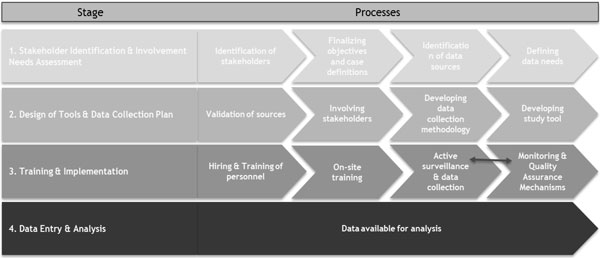
**Framework for Design and Pilot of Pak-NEDS**.

### Design and protocols

A systematic process was used to understand the needs, to design, and to initiate Pak-NEDS in the selected hospitals. The study was conducted over four months in each participating ED between November 2010 and March 2011, during which period active surveillance occurred 24 hours every day, for 7 days a week.

The sections below describe this process in more detail:

#### Needs assessment

We reviewed both published and grey literature and our search did not reveal any similar surveillance efforts implemented or underway in Pakistan. The finding was further confirmed by consultation with other emergency medicine experts, identified through the membership of the *Society of Emergency Physicians of Pakistan *(SEPP). Most of the study sites did not have adequate clinical records for clinical or research purposes, although the situation was markedly better for private institutions.

#### Stakeholder engagement

Engaging all stakeholders from the beginning of any major initiative such as this one is key to its success and sustainability [10]. Prior to the commencement of any activities, we performed a comprehensive stakeholder analysis to identify key individuals and/or departments, and understand the roles they could play in Pak-NEDS. We found that ED administrators and staff (necessary for effective coordination), and the overall hospital administration (who would be the main consumers of information such as quality of care, etc.) were key stakeholders for a successful pilot study. Public health agencies, local and national governments, and policymakers were also identified as important stakeholders, but their involvement would come after successful completion of the pilot study.

The ED administrators were identified and contacted through SEPP. One administrator from each institution was appointed as a representative of the study in the host institution. These personnel then facilitated the introduction and approval of the study by the hospital administrators and the ED staff.

The representatives were individually consulted during every stage of the surveillance design to make it compatible with the varying ED systems and protocols across the institutions, including the design and content of the standardized study questionnaire used for the data collection. A one-day ED surveillance system workshop was conducted at AKU with participants from the partner institutes. The agenda was to identify data needs that were essential to the surveillance and to identify potential sources of data already present in the EDs to minimize duplication of data collection efforts, e.g. registration information, clinical records, etc. The study data collection methods were modified accordingly and all study personnel approved the final surveillance protocols and questionnaire before the pilot testing began.

#### Study Questionnaire

Our aim was to develop an ED surveillance system model that could be replicated in other institutions and be integrated into an ED patient record form. This is reflected in the content of the questionnaire, which collected information on a wide range of issues. Table 2 outlines the different sections and lists the data points collected under each. The questionnaire was adopted from the 2010 *Emergency Department Patient Record *questionnaire of the National Hospital Ambulatory Medical Care Survey (NHAMCS) of the Centers for Disease Control and Prevention, USA [21]. It was modified, in consultation with ED administrators at participating hospitals, for compatibility with the local ED context, and to address any additional data needs. The complete questionnaire is included as Appendix A. Injury causes and presenting complaints were coded using predefined coding lists (Appendices B & C).

**Table 2:** Organization of the Pak-NEDS study questionnaire and data elements captured

Section	Data Elements
**1. Patient Personal Information**	Age; Gender; Ethnicity; Residential area; Mode of arrival; Time intervals between emergency, arrival in ED, and start of care
**2. Reason for Visit**	Presenting complaints
**3. Injuries**	Intentionality; Cause of injury; Nature of Injury
**4. History of Care**	Treatment sought from any other physician in last 72 hrs; Discharged from any hospital in last 7 days; Episode of care; Number of ED visits in past 12 months
**5. Triage**	Temperature; Pulse Rate; Respiratory Rate; Glasgow Coma Scale; Blood Pressure; Oxygen saturation; Pain Scale
**6. Diagnostic/screening Services**	Physical Examinations; Imaging; Other tests
**7. Procedures**	IV Fluids, Casts, Sutures/staples, dressing, incision & drainage, nebulized, cardiopulmonary resuscitation etc.
**8. Diagnosis**	Provisional Diagnosis; Co-morbidities; Treatment for Co-morbidities
**9. Care Providers**	Paramedics; Nurse; Clinical Intern; Post graduate trainee Physician; Consultant Physician
**10. Disposition**	Left without being seen; Follow-up planned; Return if required; Referred to other hospital; Referred to outside physician; Admitted to inpatient; etc.
**11. Total reported cost of episode of care**	In Pakistani Rupees (PKR)
**12. Discharge**	Time and Date

#### Selection of data collectors and training workshops

All data collectors involved in Pak-NEDS were required to undergo training on the study questionnaire and all relevant data collection procedures including ethical conduct during the data collection process. There was significant variation in the demographic and expertise profiles of data collectors between institutes. Priority was given to individuals who had experience in similar projects or those who had some previous training in healthcare. A number of data collectors were paramedics or nursing students. Study staff who did not have any previous clinical exposure were required to demonstrate adequate understanding of study protocols in order to continue in the project. Two-day workshops were conducted at each study site (Rawalpindi and Islamabad are sister cities, so only one workshop was conducted for both). A post-training assessment including an individual verbal assessment was completed by every team member. This step was put into place to ensure complete understanding of the study questionnaires, the components, and the potential sources of data. This was further supplemented by mock interviews conducted between the trainees to ensure accurate understanding of all questionnaire components and categories.

This was followed by an on-site one-day performance assessment for the data collection team ensuring satisfactory skills. The performance of each individual and that of the whole team was observed and assessed. Compliance with study data collection procedures, and accuracy and completeness of information collected was monitored until every member consistently demonstrated complete understanding of the data requirements of the study questionnaire, and of the sources and protocols for data collection. All trainings were conducted by AKU researchers.

#### Data Collection

**Figure 5:**
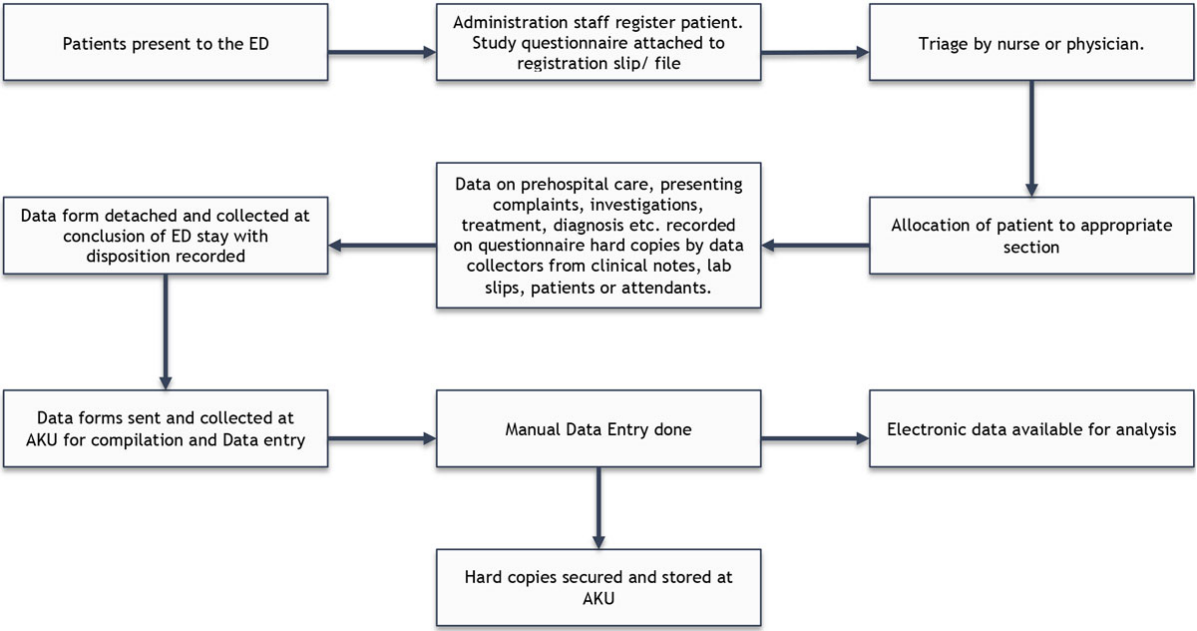
**Patient flow and data collection points at participating EDs**.

Patient flow patterns, in terms of volume and direction, were observed prior to the start of the data collection. Based on this assessment, data collectors were appointed different 'locations' within the EDs such as the triage counter, fast track clinics, critical care beds, or surgical and medical emergency rooms. These locations were different between institutions as there were differences in the distribution of services. The study questionnaire was attached to the initial registration slip or the ED record form of a patient presenting to the ED. The data collectors then proceeded to fill in the questionnaire based on information available from the clinical records, the healthcare providers, and from the patients or their attendants, in stages according to the progress of the patient within the ED until the final disposition (Figure 5). Information was not collected on clinically unstable cases until the patient or the family was available and ready to answer the questions. Questionnaires for cases who were dead on arrival or had intentional injuries were supplemented with information from their medico-legal records collected by a medical officer within the ED. Each data collector team was supervised by an on-site supervisor responsible for data quality assurance and compliance with study protocols. The supervisor was also responsible for initial data management which included the transport of the completed questionnaires to the coordinating center (AKU).

Data was collected 24 hours a day, 7 days a week in almost all of the study EDs with workers working in 8-hour shifts. Any cases which did not have a final disposition at the shift change were reported to the next shift data collectors who then followed up on those patients. Twenty-four hour coverage could not be achieved in MHL, where the number of data collectors was only sufficient to cover two 8-hour shifts every day. Weekly shifts to be covered were based on a predetermined schedule ensuring equal coverage for morning, evening, and night shifts at the end of the four-month surveillance period for MHL.

#### Data management

Study questionnaires were printed and disbursed from AKU to the other study centers. Unique IDs on each questionnaire were used to keep track of allocation and retrieval from the different sites. The on-site supervisors were responsible for sending the completed study forms to AKU (coordinating center) on a weekly basis. These forms were then entered into a computer database built using Microsoft Access. On average, computerized data was available within 10-15 days after receiving the hard copies of study forms from the field. AKU was responsible for aggregating and safekeeping the study data, including the physical forms which were catalogued and stored under lock and key in the Department of Emergency Medicine at AKU.

Individual identifying information was not collected from any of the study subjects. Confidentiality and anonymity were ensured for all individuals throughout the study. All data collected were unlinked to clinical or any other records which could be used to identify the individual patients. All electronic information was encrypted and password protected.

#### Monitoring and evaluation

A daily report of the number of cases registered in the surveillance system and those presenting to the EDs was compiled by the field supervisors and sent to AKU. A daily coverage rate (%) of the surveillance system (registered (n)/total presented (n) × 100) was calculated using this information. The total number of patients presenting at the EDs was taken from the ED registration records. These rates were used to monitor the performance of the surveillance system in terms of coverage. Any unusual deviations from the coverage trends were promptly investigated, and the reasons rectified if required.

Quality assurance processes were built in at different stages in the design and testing of Pak-NEDS (Table 3). Random checks of the completed forms (≈ 5% of total) were done in the field by the supervisors to minimize missing data, and then at the coordinating center to ensure data quality by identifying any data entry issues (such as improper formats, etc.), and any systematic errors/missing information. Feedback was provided to the relevant study supervisors for rectification of any problems.

**Table 3:** Quality assurance mechanisms employed in Pakistan National Emergency Department Surveillance

Study Stage	Quality Control Processes
**1. Stakeholder Identification & Involvement** **Needs Assessment**	Inclusion of local administrators/physicians (institution staff) as co-PIs in the study, improving local ownership and interest in proper execution of study.
**2. Design of Tools & Data Collection Plan**	Validation of data sources and data collection by site visits and on-site performance assessment. Standardized questionnaire across all centers.
**3. Training & Launch**	Thorough training including verbal tests, mock interviews, and on-site performance assessment. Field supervisors present on-site to monitor activities, ensure compliance with study protocols, and rectifying any immediate issues with support of local emergency department nominee, or reporting issues to coordinator at AKU to facilitate resolution. Random checks of forms by field supervisor of ≈5% of daily forms for completeness and accuracy Random checks at AKU for completeness of forms Feedback mechanisms for correction of any systematic errors detected in random checking. Daily reports showing total patient registrations and those covered by surveillance. Weekly report from field including coverage levels, form tracking, and any other pertinent issues affecting performance. Questionnaire tracking through ID numbers to and from centers to ensure there is no loss of data.
**4. Data Entry & Analysis**	Data entry checks with skip patterns minimizing impossible values.

#### Coverage and cost

Over 270,000 cases were registered in the surveillance system over a period of four months (Table 4). Coverage levels in the final month ranged from 91-100% and were highest in centers with the lowest volume of patients (AKU & SIH). Overall the coverage for the four months was 79%. A crude financial cost description, calculated as the total monetary cost divided by the number of ED patients captured by the system, puts the cost to obtain information from one patient using Pak-NEDS at less than US$0.20. However, it is important to note that this calculation does not take into account costs such as principal investigator time and indirect costs at the institutions where the study was implemented. In retrospect, using electronic data entry devices for data collection and investing more in supervision would have benefitted the overall study quality and ought to be factored into future costs. As such, this is an underestimate of ED surveillance costs for similar programs implemented in other settings.

**Table 4:** Pakistan National Emergency Department Surveillance Sample & Coverage

Center	Benazir Bhutto Shaheed Hospital	Lady Reading Hospital	Jinnah Post-graduate Medical Center	Mayo Hospital Lahore	Civil Hospital Quetta	Aga Khan University	Shifa International Hospital	Total
**N (row %)**	63249 (23)	59914 (21.8)	52550 (19.1)	46755 (17)	35325 (12.9)	12054 (4.4)	4589 (1.7)	274436
**Coverage (%)**								
- **Final month**	91	92	97	97	98	100	100	95.4
- **Overall**	76	79	95	89	97	90	91	79

## Discussion

Health care in Pakistan is provided by both government and private sectors; according to estimates, 70% of care is provided by private health care providers [22] This is likely to be quite the opposite for emergency care, where emergency department visits in government-run hospitals are many times higher than for the private sector. However, the true contribution of the overall private sector in emergency services is unknown [15]. The purpose of this pilot was to test the feasibility of an ED surveillance system in a low-income setting as well as understand some of the strengths, challenges, and barriers, which would be key in enabling its implementation. Therefore, while the data generated from this pilot test cannot be considered representative of the general population, its successful pilot-phase - enrolling over 270,000 people over four months with average coverage of almost eighty percent - serves as an example of how such data could be generated. If the system were to be replicated in all EDs serving the population, population-based estimates of incidence and prevalence could be retrieved from this surveillance data, and if the scale is big enough, national estimates could also be obtained [23,24]. This pilot test was done in a variety of tertiary care centers, and provides important lessons that could be applied to other similar interventions conducted in similar contexts.

Information on the occurrence and outcomes of various illnesses and injuries in Pakistan has not been available at this level before, and would be invaluable for identifying any gaps in the quality and appropriateness of services being provided in these EDs. The surveillance information could also be used to assess the effectiveness of and/or to guide improvements in interventions (e.g. changes in emergency medical services to reduce hospital transport times, physician training for improving diagnostic accuracy and treatment within the ED, modification of treatment protocols and their impacts, etc.) employed to improve healthcare services, even including those used at the community level.

ED surveillance for individual diseases has been successfully implemented in different settings. This is particularly relevant to injuries which comprise a major portion of cases presenting in EDs [10,25]. Estimates of injuries based on death certificates and trauma registries usually include only the most severe types of injuries, and community-based injury surveillance is too expensive and difficult to sustain [26,27]. ED-based road traffic injury surveillance, childhood injury surveillance, and cardiac arrest surveillance have become the formative work for ED surveillance [23,28-31].

ED databases have traditionally suffered from fragmented information coming from different sources [32]. Among the benefits of comprehensive ED-based surveillance systems is the generation of key data that can be used for evidence-based resource allocation as well as monitoring and improvement of the quality of care in EDs. This can be a strong motivating factor in implementing such a system for hospitals and even for regional health authorities. The surveillance system, if linked electronically with other hospital records, can not only reduce the burden of collecting surveillance information, but can also provide information which can be used for public health research and administrative purposes. Having standardized surveillance forms also improves comparability of findings from different institutes in such databases. The capacity for emergency medicine research is substantially increased with such a framework in place. As seen through our pilot, with minimal additional resources, additional sections or questions can be added to the basic surveillance form (if required) to answer a variety of research questions.

We identified many challenges that should be considered for similar efforts in the future (Table 5). In addition to ongoing systematic collection of information, effective surveillance systems ought to supply information in a timely manner, which can be used to address pertinent health issues. For example, the availability of real-time information could be vitally beneficial in situations where acute public health emergencies may require rapid response [6]. Due to lack of consistent data entry capacity in all centers, all forms had to be shipped to the coordinating center. With a volume of approximately 16,000 forms per week, this led to considerable delay (10-15 days) between the collection of data and its availability for analysis and use. Local data entry would result in near real-time information, and would also enable better monitoring of the data collection process. The resources and budget available for the study were limited and, in retrospect, we believe that better results in terms of coverage and data quality could be obtained by having more resources dedicated to monitoring by having more personnel, and by employment of better technology such as electronic data entry devices and their associated software and hardware. Although these devices may raise initial implementation costs of such systems, they would improve overall efficiency and potentially reduce long-term maintenance costs by circumventing the need for printed forms, data entry of these forms by separate personnel, and physical transport costs of these forms. This would also ensure real-time availability of the surveillance data and minimize errors and incomplete data, thereby improving overall data quality.

**Table 5:** Challenges and potential solutions for implementing emergency department surveillance

Challenges	Solutions
Access to institutions and EDs	Involvement of local Administrators/ED physicians as co-PIs in the study facilitated the approval process for the study from hospital administrations and institutional ethical review boards.
Overwhelming case load in public institutions Motivation of local staff to contribute	In the absence of electronic records in most cases and illegible or incomplete patient records, the data collectors had to partially rely on information from the clinical staff on the ground, especially nursing staff. Communications with the staff facilitated by the local co-PIs and relevant senior officials e.g. head nurses, about the importance and relevance of the study to them, led to limited success but this remained a challenge because of high patient volume in public institutes. One institution (LRH) offered monetary incentives to staff for participation.
Limitations of technology -No local electronic records in most cases -No mechanisms for local data entry -Free text entries -Limited information available -Limitation in obtaining real-time data as a result	Data collected on hard copies of questionnaire and transported to AKU where all data was entered on computers and then analyzed. Free text entries were limited as much as possible by extensive coding lists made available to the data collectors but they still remained a challenge.
Variations in services distribution	A standard partially modifiable data collection plan was developed in consultation with nominees and adopted according to the local context in each institute. Data collectors were appointed along major patient flow pathways within the ED to come in contact with and potentially capture the maximum number of patients presenting to the Eds.
Sustainability	Although this was a pilot phase, efforts were made to maximize future sustainability if the project continued. This involved fostering local ownership with involvement of local co-PIs and other staff, utilizing existing data sources as much as possible, and minimizing any hindrance to local staff in performance of their regular duties because of the study.
Data management logistics	All questionnaires were tracked using the study ID numbers on being shipped to and receiving from the study sites. Field supervisors were informed of the batch numbers and were responsible for safe delivery of the forms back to AKU. The ID numbers and the number of patients captured by the surveillance system were tallied with the number of returned forms at AKU using information from the daily and weekly reports.
Worker oversight and quality control	Field supervisors monitoring of data collectors at all sites and random quality checks of data. Any issues were either rectified and reported by the supervisor, or referred to the local co-PI or study coordinator at AKU for resolution. Feedback was provided to field supervisors and to data collectors regarding quality issues in the received data.

Limited and incomplete clinical records were another issue contributing to missing data in the study. The overwhelming volume of patients in some of the participating centers resulted in overburdened staff who in many cases were not able to adequately complete the clinical documentation. Almost none of the partner institutes had any electronic records other than registration information. This, coupled with illegible handwriting and misplaced forms, was a significant challenge for data collection. Despite the team's efforts to standardize coding for presenting complaints, many were recorded as free text, as were the provisional diagnoses. Abbreviations and misspellings, illegible handwriting, and data entry errors all led to these variables becoming a challenge to analyze. As mentioned earlier, the use of electronic devices in future systems would eliminate such issues.

Personnel management was another challenge in the study. One of the centers insisted on hiring their own data collectors and supervisors due to institutional policies. Although the same training and monitoring procedures were put in place as for the other institutes, coordination between the staff at this center and AKU staff was more challenging and required additional effort. The personnel that were hired at that center were government employees, which further limited control on their performance by the coordinating center. Frequent changes in personnel was another concern, which required additional trainings in some instances. These were conducted by local supervisors. In two of the public centers, ED managers who were serving as co-PIs of Pak-NEDS were locally re-assigned. Our study lasted four months and this did not have a significant impact on the pilot phase, but this could potentially be problematic for any permanent system in place requiring supervision by a specific individual. This only emphasizes the importance of ensuring local ownership of the system by the institutions themselves with protocols in place, so that any changes in the leadership do not disrupt the surveillance system.

## Conclusion

Pak-NEDS is the first comprehensive multi-center ED-based surveillance system that was developed and pilot-tested for Pakistani EDs. The system was successfully tested in a sample of the major EDs in the country, seven major tertiary care units across Pakistan having some of the highest patient volumes in the country. Through this pilot, we have shown that the system is contextually modifiable and scalable, and could potentially be adaptable for other LMIC settings.

## Ethical approval

The study was reviewed and approved by the institutional review boards of AKU and the Johns Hopkins Bloomberg School of Public Health. It was also given clearance by similar ethical approval committees at each partner site.

## Competing interests

The authors declare that they have no competing interests.

## Authors' contributions

MM was the national coordinator for Pak-NEDS. He was responsible for developing the study materials and methods, and for its pilot-testing at the national level, in collaboration with investigators from participating institutions. He wrote the first draft of the manuscript. AMB was involved in the conception of the study, analysis of the findings, and critical review and writing of the manuscript. SJ, SQA, HK, FAS, MMK, and SA were local Pak-NEDS collaborators from the participating sites. They contributed to the study design, oversaw its local activities, and critically reviewed the draft manuscript. AAH participated in the conceptualization of Pak-NEDS, and was responsible for the overall analysis plan, interpretation, and critical review of the draft. JAR was the PI of the study and was responsible for all the activities of the project conception, implementation, data collection, analysis, and manuscript review. All authors read and approved the final manuscript.
